# An increased number of individuals of a potential host facilitates non-photic synchronisation in the haematophagous insect *Triatoma infestans*

**DOI:** 10.1590/0074-02760220289

**Published:** 2023-07-31

**Authors:** Veronica Sandra Valentinuzzi/, Luciana Beatriz Abrahan

**Affiliations:** 1 Centro Regional de Investigaciones Científicas y de Transferencia Tecnológica de La Rioja, Universidad Nacional de La Rioja, Servicio Geológico Minero Argentino, Universidad Nacional de Catamarca, Consejo Nacional de Investigaciones Científicas y Técnicas, Entre Ríos y Mendoza, La Rioja, Argentina

**Keywords:** kissing bugs, non-photic cycle, biological rhythms

## Abstract

**BACKGROUND:**

*Triatoma infestans* (Kissing bug) is the main vector of the parasite causative of Chagas disease in Latin-America. This species shows clear activity rhythms easily synchronised to day-night cycles (photic cycle). The haematophagous nature of these insects lead us to think that they may temporally adapt to the particular activity rhythms of potential hosts (non-photic cycle). Our previous data showed that kissing bugs were weakly affected by the activity-inactivity rhythm of a single host.

**OBJETIVE:**

To determine if by increasing the number of individuals of a potential host, *T. infestans* could increase the likelihood of synchronisation.

**METHODS:**

Individual activity rhythms of experimental insects, maintained in constant darkness in light-tight cabinets, localised in a room with 24 rodents, were continuously monitored. Another insect group that served as control was maintained in the same conditions but in a room without rodents.

**FINDINGS:**

Most of the experimental insects synchronised, expressing a 24 h period coincident with the activity-inactivity rhythms of the rodents, while the controls free ran with a period significantly longer than 24 h.

**CONCLUSION:**

Analogous to what happens with high vs low light intensity in photic synchronisers, a high number of rodents, in contrast to the previous one-rodent experiment, increased the potency of this non-photic *zeitgeber*.

Triatomines express a regular 24-hour temporal pattern of behaviours. At the beginning of the scotophase [dark interval of a light-dark (LD) cycle], individuals search for food, engage in mating opportunities, and oviposition.^1^ ^,^ ^2^ ^,^ ^3^ ^,^ ^4^ ^,^ ^5^ At the end of the scotophase, insects return to their refuges expressing aggregation behaviour.^6^

*Triatoma infestans* (Hemiptera: Reduviidae, Klug 1838), the main vector of the causative agent of Chagas disease in Latin-America, expresses a clear nocturnal activity rhythm. This rhythm is endogenous since when exposed to constant darkness it persists with a stable period (up to 28 h) and can rapidly resynchronise when the LD cycle is restored.^7^ Because this haematophagous bug requires blood meals from living sources, an individual’s feeding and activity behaviour should reflect its assessment of the chances of obtaining food, as well as the risk involved. In nature, however, individual bugs must assess the likelihood of food availability within its habitat, suggesting that the number and concentration of potential hosts may be an important factor in synchronising the animals’ behaviour.

*Triatoma infestans* is a haematophagous bug that takes blood meals preferentially during the quiescent times of their hosts.^8^ Timing is important, not only to increase the probability of feeding, but also to avoid predation or defensive attacks from their potential host. Consequently, establishment of nearby hosts and identification of the best moment to approach and feed or avoid predation is crucial. Various host-related stimuli may be detected by the kissing bugs. The main stimuli for approaching the host are heat,^9^ ^,^ ^10^ ^,^ ^11^ CO_2_ production^12^ ^,^ ^13^ and likely the host’s activity rhythm. *T. infestans* has been reported to feed on different wild and domestic species.^14^ ^,^ ^15^ ^,^ ^16^ Therefore, as ectoparasites, we assumed that they would adapt to the particular temporal niches of different hosts. In captivity, unfed *T. infestans* show a high temporal flexibility, adapting well to daytime feeding.^17^ ^,^ ^18^ In other words, hungry kissing bugs, although in their inactive phase, and, moreover, exposed to light, move, approaching a chicken, despite this host being in its active phase.

On the other hand, free running rhythms of this same triatomine species was modulated by the daily activity of a single host (nocturnal or diurnal), housed in close proximity.^19^ These authors conducted the experiments with the bugs in constant dark, so that the synchronising signals in question were strictly non-photic. Kissing bugs’ typical long period decreased in the presence of either host with some bugs achieving relative coordination. This response may be dependent of stimulus intensity given by a single host. As has been repeatedly observed with photic cycles, low light intensity during the light phase of the LD cycle results in a less potent synchroniser. In other words, when the intensity of the light phase is increased, the contrast between light and dark is enhanced. Therefore, the LD cycle becomes a more potent synchroniser.^20^ Similarly, our hypothesis here, is that, if the number of rodents is increased, the contrast between the active phase (increased CO2, heat, and activity-generated sound)^21^ ^,^ ^22^ and the inactive phase (decreased CO2, heat and quiet sleeping behaviour)^21^ ^,^ ^22^ is amplified. This increased contrast should generate a more potent non-photic *zeitgeber*. Leaving aside the underlying mechanisms of entrainment or masking, here, we evaluated if increasing the number of rodents would increase the probability of synchronisation of *T. infestans*’ activity rhythms.

## MATERIALS AND METHODS

*Insects* - *T. infestans* same-age adult males, were maintained in the Centro Regional de Investigaciones Científicas y de Transferencia Tecnológica de La Rioja (CRILAR) colony in a controlled environment under a light:dark cycle of 12:12 h (LD; lights on at 07 h), constant temperature (27 ± 2ºC) and relative humidity (RH) (40-50%). Feeding occurred weekly on chickens (*Gallus* sp.) as part of the routine maintenance procedure. Insects were selected based on the weight considered to represent optimal health (260 mg ± 50 mg).^18^ A total of 32 insects were taken from this colony and divided in two groups of 16 each. The experimental group was localised in the animal room that lodged 24 rodents. The control group was maintained in a different room with no nearby host, as explained below.

*Insects recording system and housing* - The selected insects were fed on chickens, as usual, and immediately individually housed in monitoring units for activity recording, as in Valentinuzzi et al.^7^ Once they were placed in these units, they remained undisturbed for the whole 33-day recording interval (not even feeding occurred). Each individual recording unit was made of a rectangle base of thin balsa wood (3x10 cm) with a semi cylindrical nylon-netting top (3.5 cm high). A wire across the width of the base at the longitudinal midpoint allowed each cage-like unit to pivot on two lateral cradle-like supports. A 15-cm copper wire, longitudinally placed along the base, could contact with an external wire on each side, according to the pivoting position of the cage. As the insect inside walked across the mid line of the cage, the actimeter would slightly re-balance (as a seesaw), closing an electric circuit at each inclination. Each electric contact was registered as a pulse of activity. Locomotor activity was recorded continuously every 5-minute interval using Vital-View (Phillips-Respironics, Bend, OR^®^).

**Fig. 1:**
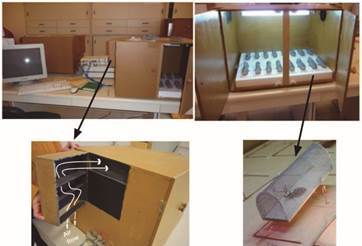
kissing bug monitoring system. Upper photographs show one of the isolating wooden cabinets with doors open (right) and partially closed (left). Below-right is an individual monitoring unit and below-left a disassembled light-trap box. Parallel and alternated wooden sheets (similar to a muffler exhaust) allow air entrance (flow direction shown by white arrows), while avoiding light. The black painting in the inside further avoids light entrance. A digital Luximeter Model TM-201 (Tenmars Electronics CO., Ltd, Taiwan) was used to confirm total darkness inside the cabinets when doors are closed and fluorescent light bulb off.

These monitoring units were distributed in two light-tight ventilated wooden cabinets (60x40x45 cm). The ventilation system of these cabinets assured the continuous circulation of air from the outside room into the cabinet, and out again to the room while avoiding light entrance through a light trap system (Fig. 1). The two cabinets were positioned in two different rooms. The cabinet with the 16 experimental insects was placed in the animal room that lodged 24 rodents. The cabinet with the 16 control insects was localised in another room without any potential host.

*Vertebrate used as the non-photic cycle* - Twenty-four subterranean rodents (*Ctenomys* sp), commonly known as tuco-tucos were used, each weighting 120 to 240 g. This species was selected based on five reasons: (a) Due to years of study of our lab, we understand their temporal organisation fairly well. All animals show clear and robust activity rhythms,^23^ and more importantly, they are nocturnal in the lab but diurnal in the field;^*(e.g.,24)*^ (b). Our Lab has a colony in which individual activity is constantly recorded, a fact that strongly facilitated the logistics of the present experiment; (c) In the field, diurnal tuco-tucos^24^ share the same habitat with nocturnal *T. infestans*; (d) In Lab, fasted kissing bugs have been monitored feeding on tuco-tucos during the light phase (Abrahan L and Tachinardi P, unpublished observations); (e) In the laboratory setup, the daily activity of a single tuco-tuco in close proximity, has been shown to modulate the activity of triatomines.^19^

Rodents were individually housed in running-wheel cages in the animal room, and their wheel-running activity rhythms were used as the marker of the potential non-photic synchroniser. The activity rhythms of the 24 rodents were clearly synchronised to the natural photoperiod (through a glass window). Since tuco-tucos maintained in the lab are nocturnal,^24^ their activity was concentrated during the dark phase. Temperature was registered at 15-minute intervals with data loggers (HOBO data loggers U1P0/003 Onset Computer Corporation, Bourne, MA^*®*^ ), verifying that values were maintained stable in 24 ± 1ºC.

*Data analysis* - The activity rhythms of each kissing bug were depicted in double-plotted actograms using the software *El Temps* (A. Díez-Noguera, Universitat de Barcelona, 1999). Actograms allowed visual estimation of phase and rhythmic patterns. Analysis of actograms were done through visual inspection of two independent observers, assisted by the “point-and-read” tools of *El Temps*. Activity onset was determined as in Valentinuzzi et al.,^7^ defined as the first bout of activity of at least 60 minutes of continuous activity after a previous 60-min or longer interval of inactivity. Importantly, this definition had to be in accordance with a group pattern, that is, this had to occur for a minimum of two consecutive days, and in a coherent way (showing a 24 h period related to synchronisation or a consistently increasing or decreasing period in case of constant conditions). Synchronisation or free-run was determined estimating period of each experimental and control individual, using the original Chi-square periodogram analysis, described by Sokolove and Bushell^25^ with a global risk level of p ≤ 0.05. Number of days it took to reach synchronisation in experimental insects, as well as the calculated period values for both groups, were expressed in mean values ± standard error. When comparing both groups, an unpaired t-test was used in excel. In the actograms, the non-photic cycle was the wheel-running rhythms of the rodents, represented as the mean value of the 24 animals.

*Ethics* - Chicken maintenance was approved and authorised by the Experimental Ethics Committee, of the Public Health State Ministry of La Rioja Province, Argentina (Permit # 892). *Ctenomys* maintenance in the laboratory was authorised by the Secretaría de Ambiente-Ministerio de Planeamiento e Industria de La Rioja (Expte. Nº P4 00501-17-2018), and approved by the Ethics Committees of the Faculty of Veterinary Sciences of La Plata National University (Permit 29-2-12).

## RESULTS

**Fig. 2:**
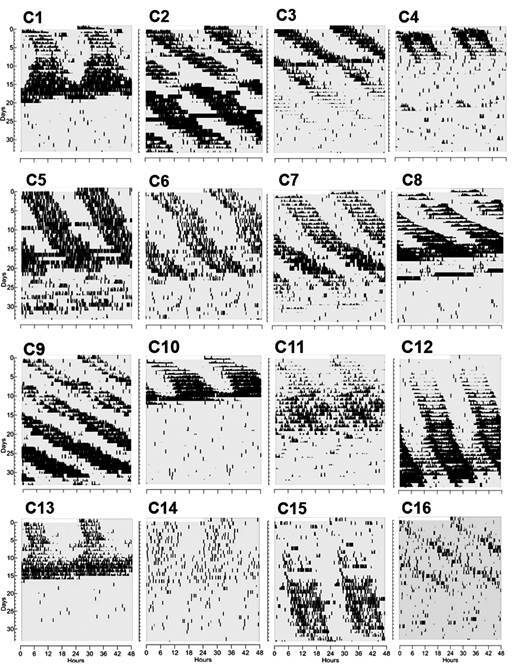
individual actograms of the 16 control insects that remained undisturbed in constant darkness (grey background). Days are represented in the left axis and a 48-hour scale in the abscissa. Black marks represent activity of the insects.

In control insects, actograms in constant darkness showed clear free-running rhythms, most of them very robust (Fig. 2). Two control animals, C12 and C15, took a while to manifest this robustness but did so on days four and ten, respectively. Of the 16 control insects, four (C2, C9, C12 and C15) continued to manifest their free-run until day 33 when recording was halted. In the remaining 12 insects, as time elapsed, arrhythmic patterns became the common output. This arrythmicity was initiated around mean day 18.4 ± 1.3 and continued until the end of the recording interval. Periodograms revealed periods longer than 24h with a mean value of 25.5 ± 0.2 h.

**Fig. 3:**
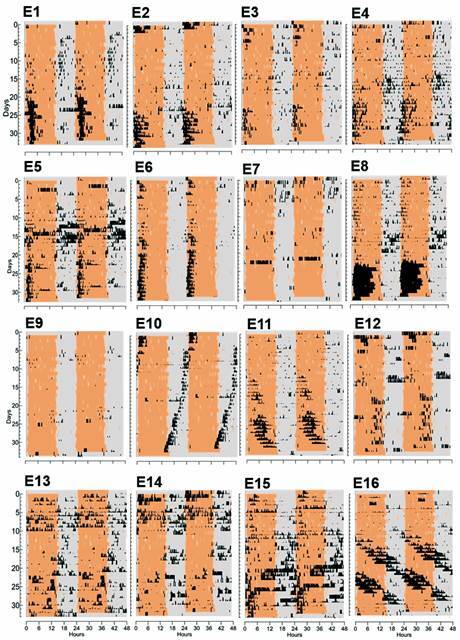
individual actograms of the 16 experimental insects maintained in constant darkness (grey background) and housed in the animal room with 24 rodents. Days are represented in the left axis and a 48-hour scale in the abscissa. Black marks represent activity of the insects and the orange background, mean activity of the 24 rodents.

The actograms of the insects from the experimental group showed a totally different pattern (Fig. 3). Not only did they not express a free-running rhythm, but most of them synchronised to the rodent’s non-photic cycle at some point of the monitoring interval. Of the 16 insects, nine (E1, E2, E3, E4, E5, E8, E11, E12, E15) exhibited synchronisation to the non-photic cycle after 16 to 24 days [mean ± standard error (SE) was 20.4 ± 0.9 days] of exposure to the rodents, adjusting their active phase to the host’s wake phase. At the same time, one insect (E6) did the same, but from the beginning to the end, remaining synchronised along the whole recording interval. During these synchronised intervals, periodograms revealed that the experimental insects had periods equal to 24 h in accordance with synchronised individuals. With respect to the remaining six insects of the experimental group, two (E7, E9) never showed enough activity despite remaining alive until finalisation of the experiment. Another two (E10, E16) appeared to ignore the non-photic stimuli since they showed free-running rhythms. Finally, E13 and E14 were mainly arrhythmic with only a certain degree of messy activity concentrated during the rodent’s rest phase (on days 11 to 22 for E13 and days 15 to 19 for E14).

When comparing the period between free-running controls (n = 16) and synchronised experimental insects (n = 11), the difference was very significant (p < 0.001).

## DISCUSSION

The data show that, when kissing bugs are exposed to a high number of individuals of a potential host, they are capable of adjusting their own daily activity rhythms to the activity rhythm of the group of rodents. This fact suggests a high potency of the non-photic synchroniser. Of the 16 experimental insects, ten clearly synchronised to the rodent’s rhythm expressing a 24 h period, in contrast to the free-run of all control insects with tau values significantly longer than 24 h. As mentioned above, leaving aside any type of endogenous mechanism, this phenomenon could be considered analogous to what happens when the contrast between the light phase and the dark phase of a photic cycle is increased: its synchronising potency is augmented.^20^

This high-number rodent result, contrasts with the one-rodent experiment of Lopez et al.,^19^ where in the latter, insects only showed modulation of their rhythmicity, reinforcing the idea that more rodents may imply in a stronger synchroniser. Domestic triatomine species tend to be more present in rural dwellings with a higher proportion of humans and/or domestic animals (chickens, goats, dogs, etc.) attracted by a larger biomass.^26^ In other words, exposure to the activity-inactivity cycles of a high number of individuals of a potential host may be a more natural condition for this haematophagous species. The rodent species used here shows an intermediate form of sociability, characterised by persistent partial home range overlap, determined by radiotelemetry in the field.^27^ We acknowledge that it is unknown if *Ctenomys* is a natural host for kissing bugs. However, we have witnessed in Lab settings that fasted kissing bugs approach and feed on tuco-tucos during the light phase (Abrahan and Tachinardi, unpublished observations).

Despite these observations, it is a fact that the biomass offered to the insects in the present work is larger than that in Lopez et al.,^19^ and consequently, more pronounced rodent signals are likely being emitted, increasing the probability of kissing bugs’ perception. As mentioned above, these signals could be CO2 and heat, both signals that upsurge during the active phase of the rodents. Another possible signal could be the intensification of sounds produced by rodents while in their active phase. For instance, tuco-tucos commonly express lousy intense excavation movements on the cage floors, or shovelling the wood ships as well as food around the cages, grooming, and other general sound-generating movements. All these signals could easily reach the kissing bugs through the ventilation system of the light-tight cabinets in which they were housed.

Even though, there is a tendency to consider that insect’s activity in this setting represents motivation to find food and that this preferentially occurs during the rodents’ rest phase, here we see the opposite. That is, when synchronisation happens, insect’s activity is concentrated during the active phase of the rodents. As mentioned in the Introduction section, in the vivarium, kissing bugs are regularly fed on chickens.^17^ ^,^ ^18^ This procedure occurs normally, despite the chickens being awake, despite exposure to light, and despite the fact that the insects are in their inactive phase. The capacity of an organism to be active in either day or night, depending on prevailing environmental conditions, is viewed as adaptive and indicate a high degree of phenotypic plasticity.^28^ Importantly, locomotor activity displayed by an animal under laboratory conditions may - regardless of the measurement method - represents the expression of different behavioural programs under natural conditions. These behaviours may be foraging, mate searching or escape actions.^29^

An interesting and unexpected result was the arrhythmicity observed in 12 of the 16 control insects. This occurred around days 18 to 20, in strong contrast with the experimental insects that, by the same time, were not only more active but also rhythmic. Furthermore, most of them were well synchronised to the non-photic cycle. An issue that may be of interest to consider is that, as the monitoring interval of both groups continued, the fasting interval in the insects increased. Recall that their last feeding occurred before introducing the insects in their recording units. Interestingly, both control and experimental groups showed these drastic changes at the same time interval (Controls n = 12, 18.4 ± 1.3 vs Experimentals n = 9, 20.4 ± 0.9 days; t-test p = 0.1). That is, the controls became arrhythmic while the experimentals became synchronised around days 18 to 20. This time lapse represents almost half of the 55-day maximum interval of fasting that an adult kissing bug can support before deceasing.^18^ Lehane and Schofield^30^ reported that active dispersion in *T. infestans* depends on nutritional status, being 12-18 days of fasting a breakpoint in insects. In the present work, our interpretation for the control and the experimental groups is the following. At this same fasting interval, activity increase in the control insects may be in an attempt to mobilise and find food. However, later on, and with no indication of the proximity of food, it may have become more adaptive to decrease or even abolish activity in order to save energy. In the experimental group, the detection of the rodents may be leading to a totally opposite behaviour, characterised not only by increased activity, but also rhythmic expression, and more on, synchronised to the potential food source and/or predator. This hypothesis should be tested by further experiments.

*In conclusion* - Kissing bugs can synchronise to potential hosts. The strength of the entrainment will depend on the potency of the non-photic cycle that can be amplified by increasing the number of potential hosts. This is analogous to what happens with low light intensity vs high light intensity when a photic cycle is the synchroniser.
